# Neuroprotection by Transcranial Direct Current Stimulation in Rodent Models of Focal Ischemic Stroke: A Meta-Analysis

**DOI:** 10.3389/fnins.2021.761971

**Published:** 2021-11-23

**Authors:** Jiapeng Huang, Kehong Zhao, Ziqi Zhao, Yun Qu

**Affiliations:** ^1^Department of Rehabilitation Medicine, West China Hospital, Sichuan University, Chengdu, China; ^2^Key Laboratory of Rehabilitation Medicine in Sichuan Province, West China Hospital, Sichuan University, Chengdu, China; ^3^Research Laboratory of Neurorehabilitation, Research Institute of Rehabilitation Medicine, West China Hospital, Sichuan University, Chengdu, China

**Keywords:** transcranial direct current stimulation, ischemic stroke, rodent model, cerebral infarction, meta-analysis

## Abstract

Infarct size is associated with stroke severity in clinical studies, so reducing it has become an important target and research hotspot in the treatment of ischemic stroke. Some preclinical studies have shown transcranial direct current stimulation (tDCS) reduced infarct size and improved neurological deficit, but others have not found beneficial effects. Besides, the optimal pattern of tDCS for ischemic stroke remains largely unknown. To shed light on the current circumstance and future research directions, the systematic review evaluated the effect of different tDCS paradigms in reducing infarct size and improving neurological deficit in rodent models of ischemic stroke and assessed the methodological quality of current literature. We searched the MEDLINE (*via* PubMed), EMBASE, Web of Science, and Scopus from their inception to August 18, 2021, to identify studies evaluating the effects of tDCS in rodent models of ischemic stroke. Eight studies were included, of which seven studies were included in the meta-analysis. The results showed cathodal tDCS, rather than anodal tDCS, reduced infarct size mainly measured by tetrazolium chloride and magnetic resonance imaging (standardized mean difference: −1.13; 95% CI: −1.72, −0.53; *p* = 0.0002) and improved neurological deficit assessed by a modified neurological severity score (standardized mean difference: −2.10; 95% CI: −3.78, −0.42; *p* = 0.01) in an early stage of focal ischemic stroke in rodent models. Subgroup analyses showed effects of cathodal tDCS on infarct size were not varied by ischemia duration (ischemia for 1, 1.5, and 2 h or permanent ischemia) and anesthesia (involving isoflurane and ketamine). The overall quality of studies included was low, thus the results must be interpreted cautiously. Published studies suggest that cathodal tDCS may be a promising avenue to explore for augmenting rehabilitation from focal ischemic stroke. Considering the methodological limitations, it is unreliable to blindly extrapolate the animal data to the clinical practice. Future research is needed to investigate the mechanism of tDCS in a randomized and blinded fashion in clinically relevant stroke models, such as elderly animals, female animals, and animals with comorbidities, to find an optimal treatment protocol.

## Introduction

Stroke, a leading cause of mortality, leads to over two million new cases annually and is associated with the highest disability-adjusted life-years lost of any disease in China (Guan et al., 2017; Wu et al., 2019). Approximately 80% of all strokes result from ischemic stroke (Moretti et al., 2015). As a cardinal outcome of ischemic stroke, large infarct size may contribute to the death in the first month following stroke and poor functional outcomes in clinical studies (Laredo et al., 2018) and may make it difficult for patients to recover from stroke once irreversible damage occurs. Therefore, reducing infarct size should be an important part of ischemic stroke treatment.

Recently, transcranial direct current stimulation (tDCS) as a non-invasive, easy to administer, safe, and well-tolerated technique has received growing interest owing to its potential efficacy in modulating plasticity in healthy persons and patients (Beaulieu et al., 2019; Machado et al., 2019). Non-invasive tDCS is the process of delivering a weak electric direct current through the scalp to benefit from its cortical excitability modifying effect. It modulates cortex excitability mainly by affecting the membrane polarity. While anodal stimulation which places the anode electrode near the target area is considered to induce neuronal membrane depolarization and increase cortical excitability, cathodal stimulation is presumed to induce hyperpolarization and reduce cortical excitability (Nitsche and Paulus, 2001; Nitsche et al., 2003b). And an interhemispheric rivalry model between the damaged and the intact hemispheres provides a framework for tDCS application, which upregulates the excitability of the affected hemisphere cortex through anodal tDCS and downregulates the excitability of the unaffected hemisphere cortex through cathodal tDCS (Bolognini et al., 2011; Rocha et al., 2016). Regarding the stimulation locus, primary motor cortex (Yoon et al., 2012; Viana et al., 2014; Andrade et al., 2017), premotor cortex (Andrade et al., 2017), and primary sensorimotor cortex (Qu et al., 2009; Wu et al., 2013) were involved in most studies with tDCS. Neural networks within the central nervous system have plasticity following stroke, and tDCS may play a potential therapeutic role by changing an eventual maladaptive pattern of activation and *via* the production of long-term important changes in brain plasticity (Beaulieu et al., 2019; Bucur and Papagno, 2019). The mechanisms underlying tDCS treatment may involve changes in the activity of the Na^+^/Ca^++^channel, *N*-methyl-D-aspartate receptor, brain-derived neurotrophic factor, and tropomyosin receptor kinase B (Liebetanz et al., 2002; Nitsche et al., 2003a; Fritsch et al., 2010; Takebayashi et al., 2017). Besides, the effect of tDCS may be related to the molecular mechanisms of promoting ischemic tolerance, neuroprotection, neurogenesis, angiogenesis, and anti-apoptosis, which may reduce inflammation, edema, or infarct size and improve neurological deficit following ischemic stroke. Previous studies have investigated the effect of tDCS on infarct size and neurological deficit, but results have been inconsistent (Kim et al., 2010; Notturno et al., 2014). Furthermore, the rationale for using different paradigms is rarely justified and there is a lack of consensus on the standardized paradigms and protocols for the use of tDCS for ischemic stroke. Besides, no meta-analysis has evaluated the neuroprotective effect of tDCS following ischemic stroke.

In the present meta-analysis, we focus on different tDCS paradigms, with the primary objective being to evaluate the effect of different tDCS paradigms in reducing infarct size and improving neurological deficit from focal ischemic stroke in rodent models. Our second objective was to examine if the effects of tDCS were influenced by the duration of ischemia and anesthesia used in intervention procedures. Our third objective was to shed light on knowledge gaps in the preclinical tDCS research literature by evaluating its risk of bias and explore the possibility of whether the results obtained from these animal studies may be helpful in designing future animal studies on the effect of tDCS in the treatment of ischemic stroke.

## Methods

This meta-analysis was performed in line with the Cochrane Collaboration (Johnson et al., 2004) and the Preferred Reporting Items for Systematic Review and Meta-analyses (PRISMA) guidelines (Fan et al., 2017; Supplementary Material 1). Our protocol was registered in the International Platform of Registered Systematic Review and Meta-analysis Protocols database under the number INPLASY202150080. As all analyses were based on previously published studies, no ethical approval was needed.

### Search Criteria

The final literature search was completed on August 18, 2021, to identify studies evaluating the effects of tDCS in rodent models of ischemic stroke, using the following electronic bibliographic databases: MEDLINE (*via* PubMed), EMBASE, Web of Science, and Scopus. The search string was built as follows: individually or combined included stroke, tDCS, muridae, and a string of words that were determined after multiple pre-searches (Supplementary Material 2).

### Study Selection

The screening was performed in two phases, namely initial screening based on title and abstract, followed by a full-text screening of the eligible articles for final inclusion. In each phase, two observers independently assessed each article. Discrepancies were resolved through discussion, or by consulting a third investigator. Inclusion criteria were: (1) Preclinical studies using rodents were required to establish a focal ischemic stroke model, regardless of the modeling method, and receive tDCS with unlimited polarity, current density, duration, and timing of application. (2) Controlled studies with a separate control group, which received sham tDCS or blank treatment. (3) Studies had to provide data on cerebral infarct size, regardless of the method of evaluation, which can be tetrazolium chloride (TTC), magnetic resonance imaging (MRI), cresyl violet, etc. And infarct size can be expressed as a percentage of the hemisphere, percentage of the whole brain, in cm^2^, or mm^3^. Exclusion criteria were: (1) Review, editorial, conference abstract, and non-English publications. (2) Studies using rodent models of global ischemia or hemorrhage stroke, and those using non-rodent models, *ex vivo* and *in vitro* preparations, or humans. (3) TDCS had no definite anodal and cathodal electrodes and was used in combination with another treatment. (4) Studies without a separate control group. (5) No model control group that did not receive tDCS. Authors were contacted to provide additional information *via* email in cases of ambiguity.

### Data Extraction and Quality Assessment

Two independent reviewers independently extracted data from the text and Supplementary Materials, or from figures using Engauge Digitizer when no data was explicitly reported. The data included infarct size, neurobehavioral outcomes evaluated by a modified neurological severity score (mNSS), mortality, and adverse events. As the missing data has not been peer-reviewed, we did not contact the authors to provide it. Species, sex, weight, age, modeling methods, ischemic duration, experimental groups, control group(s), number of animals per group, methods used to assess the outcome, type of stimulation, stimulation locus, current density, intervention duration, the timing of intervention, and anesthesia used for the intervention procedure were also extracted. We resolved discrepancies through discussion, or by consulting a third investigator.

Two investigators independently read the included literature and assessed the risk of bias. The SYRCLE animal experiment bias risk assessment tool was applied to evaluate the risk of bias in individual included studies (Hooijmans et al., 2014b). We resolved discrepancies through discussion, or by consulting a third investigator. Studies were divided into low-bias risk, high-bias risk, and unclear bias risk.

### Data Analysis

Meta-analysis was performed using Review Manager (RevMan) software (The Cochrane Collaboration, version 5.3). For continuous variables, a standardized mean difference (SMD) was calculated using random-effects inverse variance meta-analyses and presented with 95% confidence intervals if measurement methods were different among the included studies; otherwise, a mean difference (MD) was calculated. Because of the exploratory nature of animal studies, a random-effects model was used to account for anticipated heterogeneity. To avoid double-counting control animals, control group sample sizes were split in case of studies using multiple experimental groups and a single control group. The *I*^2^ was used for evaluating heterogeneity. Where necessary data were available, we performed subgroup analyses to examine whether the effect of tDCS varied by the duration of ischemia and anesthesia used for the intervention procedure. Leave-one-out sensitivity analyses were performed to evaluate the robustness of the results. If there were 10 or more articles included in a certain index, a funnel plot was used to analyze the publication bias; otherwise, the publication bias would not be analyzed. The results of the meta-analysis were presented using forest plots. If meta-analysis was not possible, data was reported through a descriptive summary.

## Results

### Characteristics of Included Studies

**FIGURE 1:**
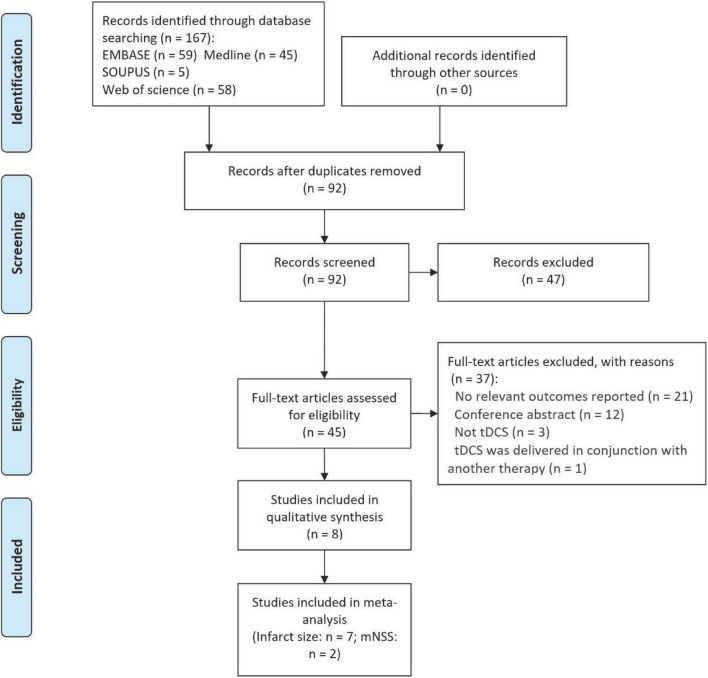
PRISMA flow diagram for search strategy and study selection.

A total of 167 potentially eligible studies were identified by the initial database search. After duplicate removal and title-abstract screening, 45 studies were selected to determine their eligibility. After excluding 37 studies, eight studies (Kim et al., 2010; Yoon et al., 2012; Peruzzotti-Jametti et al., 2013; Notturno et al., 2014; Braun et al., 2016; Zhang K. et al., 2020, Zhang K. Y. et al., 2020; Cheng et al., 2021) were included, of which seven studies (Kim et al., 2010; Yoon et al., 2012; Peruzzotti-Jametti et al., 2013; Notturno et al., 2014; Braun et al., 2016; Zhang K. et al., 2020, Zhang K. Y. et al., 2020) were included in the qualitative synthesis (Figure 1 and Table 1). Included studies were published between 2010 and 2021. Samples sizes in the included studies ranged from 6 to 24. In terms of the species used in each study, seven studies (Kim et al., 2010; Yoon et al., 2012; Notturno et al., 2014; Braun et al., 2016; Zhang K. et al., 2020, Zhang K. Y. et al., 2020; Cheng et al., 2021) employed rats, and one study (Peruzzotti-Jametti et al., 2013) used mice (Table 1). Male animals were the most common animal for strokes (Yoon et al., 2012; Peruzzotti-Jametti et al., 2013; Notturno et al., 2014; Braun et al., 2016; Zhang K. et al., 2020, Zhang K. Y. et al., 2020; Cheng et al., 2021), while one study (Kim et al., 2010) did not specify the animal sex. Permanent models were induced in two studies (Kim et al., 2010; Notturno et al., 2014) and temporary models were induced in six studies (Yoon et al., 2012; Peruzzotti-Jametti et al., 2013; Braun et al., 2016; Zhang K. et al., 2020, Zhang K. Y. et al., 2020; Cheng et al., 2021).

**TABLE 1:** Characteristics of studies included in this meta-analysis, *K* = 7.

Study	Rodents used	Age (weeks)	Duration of ischemia	Protocol of tDCS	Parameters of tDCS	Control intervention used for this review	Timing of intervention relative to stroke induction	Anesthesia used during intervention procedures
Braun et al., 2016	Male Wistar rats (body weight 290–330 g)	–	1 h	15 min, 1 time daily, take a rest for 2 days after 5 days treatment, and then for 5 more days	Anodal and cathodal stimulation; 500 μA; 128,571 C/m^2^; bregma AP + 2.0 mm, ML + 2.0 mm	Sham tDCS	3 days after ischemia	Isoflurane
Kim et al., 2010	Sprague-Dawley rats (body weight 290–330 g)	5	Permanent	30 min, once a day for 2 weeks	Anodal and cathodal stimulation; 100 μA; 3 mm to the left and 2 mm in front of the interaural line	No treatment	2 days postoperatively	1% ketamine (15 mL/kg)
Notturno et al., 2014	Male Sprague-Dawley rats (body weight not specified)	8–9	Permanent	One session: 4 and 6 h (alternating15 min on and 15 min off)	Cathodal stimulation; 200 μA; 2.86 mA/cm^2^; l 2 mm left and 1 mm posterior to the bregma	Sham tDCS	45 min after ischemia; soon after ischemia	2% isoflurane
Peruzzotti-Jametti et al., 2013	Male C57BL/6 mice (20–22 g)	8–10	1.5 h	One session: 20 min, followed by 20 min rest and additional 20 min tDCS	Anodal and cathodal stimulation; 250 μA; 5.5 mA/cm^2^; 2.5 mm left and 0.5 mm posterior to the bregma	Sham tDCS	starting 30 min (or 4, 5 h) after ischemia	–
Yoon et al., 2012	Male Sprague-Dawley rats (body weight 220–280 g)	6	2 h	20 min, once a day for 5 days	Anodal stimulation; 200 μA; 2.82 mA/cm^2^; M1	Sham tDCS	1 day or 1 week after ischemia	2% isoflurane
Zhang K. et al., 2020	Male Sprague-Dawley rats (body weight not specified)	–	Temporary (not specified)	15 min, once a day, 5 days, followed by 2 days rest and additional 5 days tDCS	Cathodal stimulation; 500 μA; 128,571 C/m2; bregma AP + 2.0 mm and ML + 2.0 mm	Sham tDCS	2 days after ischemia	None
Zhang K. Y. et al., 2020	Adult male Sprague-Dawley rats (230–250 g)	–	Temporary (not specified)	15 min, once a day, 5 days, followed by 2 days rest and additional 5 days tDCS	Cathodal stimulation; 500 μA; bregma AP + 2.0 mm and ML + 2.0 mm	Sham tDCS	2 days after ischemia	None
Cheng et al., 2021	Adult male Sprague-Dawley rats (230–250 g)	–	1.5 h	10 min, followed by 3 min rest and then 10 min stimulation, for a total 8 times of 10 min stimulation	Dual stimulation; 100 μA; of 2.86 mA/cm^2^	Sham tDCS	3 h after ischemia-reperfusion	None

*tDCS, transcranial direct current stimulation.*

One study (Yoon et al., 2012) used solely anodal tDCS, three studies (Notturno et al., 2014; Zhang K. et al., 2020, Zhang K. Y. et al., 2020) used solely cathodal tDCS, three studies (Kim et al., 2010; Yoon et al., 2012; Peruzzotti-Jametti et al., 2013) used both types of tDCS as an intervention, and one study (Cheng et al., 2021) used dual tDCS (Table 1). Variations in the stimulation locus, density of current, intervention duration, and timing of intervention were observed. Of the included studies, anesthesia was administrated in four studies (Kim et al., 2010; Yoon et al., 2012; Notturno et al., 2014; Braun et al., 2016), three studies (Zhang K. et al., 2020, Zhang K. Y. et al., 2020; Cheng et al., 2021) explicitly stated the animals were kept awake, and one study (Peruzzotti-Jametti et al., 2013) did not report whether anesthesia was used during the tDCS procedure (Table 1). Of the included studies, most of the studies did not report the measurement of temperature (Kim et al., 2010; Notturno et al., 2014; Braun et al., 2016; Zhang K. et al., 2020), three studies (Yoon et al., 2012; Peruzzotti-Jametti et al., 2013; Cheng et al., 2021) maintained the temperature, and one study (Zhang K. Y. et al., 2020) explicitly stated that temperature was not measured during the tDCS procedure. Regarding the method of infarct size evaluation, TTC staining was the most common method for infarct size assessment (Kim et al., 2010; Zhang K. et al., 2020, Zhang K. Y. et al., 2020; Cheng et al., 2021), followed by MRI (Yoon et al., 2012; Braun et al., 2016), cresyl violet (Notturno et al., 2014), and Fluoro-Jade B (Peruzzotti-Jametti et al., 2013; Table 2).

**TABLE 2:** Characteristics of outcome evaluations, *K* = 7.

	Study	Infarct size			Neurological deficit		
			
		Method	Timing	Reported outcome	Method	Timing	Reported outcome
Permanent ischemia	Kim et al., 2010	TTC	16 days postoperatively	**↔**	–	–	–
	Notturno et al., 2014	Cresyl violet	48 h after ischemia	**↓**	–	–	–
Temporary ischemia	Braun et al., 2016	MRI	2 days after ischemia	**↔**	–	–	–
	Peruzzotti-Jametti et al., 2013	Fluoro-Jade-B	24 or 72 h after ischemia	**↑↓↔**	mNSS	24 or 72 h after ischemia	**↑↓↔**
	Yoon et al., 2012	MRI	1 day, 2 weeks, or 4 weeks after ischemia	**↔**	–	–	–
	Zhang K. et al., 2020	TTC	3 days after ischemia	**↓**	–	–	–
	Zhang K. Y. et al., 2020	TTC	3 days after ischemia	**↓**	mNSS	2, 4, 6, 8, 10, 12, and 14 days after ischemia	**↓**
	Cheng et al., 2021	TTC	24 h after ischemia	**↓**	mNSS	1, 3, 7, 14 days after ischemia	**↓**

*TTC, tetrazolium chloride; MRI, magnetic resonance imaging; mNSS, modified neurological severity score;**↔** no statistically significant difference between groups;**↓** Significant decreased in mice receiving tDCS;**↑** significantly improvement in animals receiving tDCS.*

The effect of dual tDCS on infarct size and mNSS was assessed in only one study (Cheng et al., 2021) and therefore meta-analyses relating to this outcome were not conducted. This study indicates that dual tDCS can reduce the infarct size at 24 h and promote functional recovery after ischemia-reperfusion.

### Quality Assessment

Based on the SYRCLE animal experiment bias risk assessment tool, we found the overall quality of the studies was low (Supplementary Material 3). All the studies did not adequately generate the allocation sequence nor describe the random component in this process. Similarly, all the studies did not describe the method used to conceal the allocation sequence. Only two studies described that animals were studied in a blinded fashion for treatment. Similarly, only two studies reported that animals were selected randomly for outcome measurement, but both of them did not describe the random component in this process. Most studies did not explicitly describe if all animals were included in the analysis and two studies did not report if the death of animals influenced the true outcome. Five studies reported ensuring that the housing conditions were identical, while the other three omitted to describe if animals were housed identically during the experiment. Five studies reported that the groups were similar before tDCS. Seven studies described that the outcome assessor was blinded. All studies had an unclear risk of bias regarding other sources of bias since it was not clear whether brain slices were selected randomly for infarct size measurement.

### Meta-Analyses on Infarct Size

#### The Effect of Anodal Transcranial Direct Current Stimulation on Infarct Size

**FIGURE 2:**
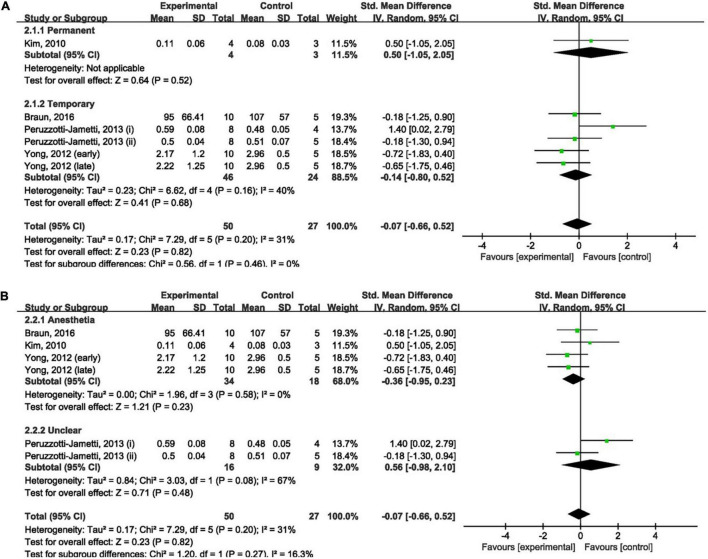
Forest plot analysis of the efficacy of anodal tDCS on infarct size compared to controls divided on: **(A)** duration of ischemia; **(B)** anesthesia used during tDCS procedure.

Four studies (Kim et al., 2010; Yoon et al., 2012; Peruzzotti-Jametti et al., 2013; Braun et al., 2016) measured the effect of anodal tDCS on infarct size and were included in our meta-analysis. Meta-analysis showed that anodal tDCS could not reduce infarct size when compared to the control groups (SMD: −0.07; 95% CI: −0.66, 0.52; *p* = 0.82; *I*^2^ = 31%; Figures 2A,B).

The duration of ischemia was used to divide the subgroups. No significant difference was found between permanent (SMD: 0.50; 95% CI: −1.05, 2.05; *p* = 0.52; Figure 2A) and temporary (SMD: −0.14; 95% CI: −0.80, 0.52; *p* = 0.68; *I*^2^ = 40%; Figure 2A) models when compared to the control group. Leave-one-out sensitivity analyses showed no difference in the overall finding that anodal tDCS did not reduce infarct size (Supplementary Material 4A).

The anesthesia used during the tDCS procedure was employed to divide the subgroups. There was no significant difference in the effect of anodal tDCS between studies that used anesthesia (SMD: −0.36; 95% CI: −0.95, 0.23; *p* = 0.23; *I*^2^ = 0%; Figure 2B) and studies that did not report the use of anesthesia (SMD: 0.56; 95% CI: −0.98, 2.10; *p* = 0.48; *I*^2^ = 67%; Figure 2B) when compared to the control group. Exclusion of any single study showed no difference in the overall finding that anodal tDCS did not reduce infarct size (Supplementary Material 4B).

#### The Effect of Cathodal Transcranial Direct Current Stimulation on Infarct Size

**FIGURE 3:**
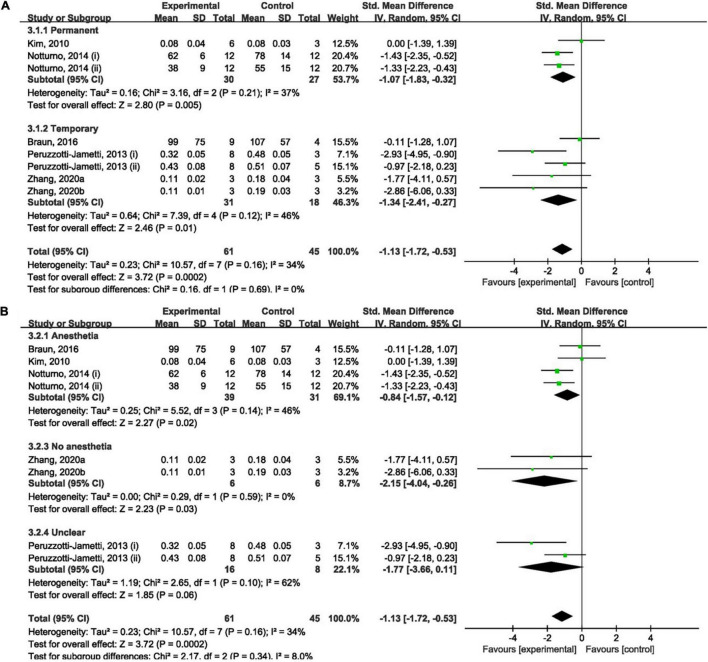
Forest plot analysis of the efficacy of cathodal tDCS on infarct size compared to controls divided on: **(A)** duration of ischemia; **(B)** anesthesia used during tDCS procedure.

Overall, cathodal tDCS was suggested to have a positive effect by reducing infarct size (Figures 3A,B). Cathodal tDCS groups significantly reduced infarct size when compared to the control groups (SMD: −1.13; 95% CI: −1.72, −0.53; *p* = 0.0002; *I*^2^ = 34%).

No significant difference between permanent (SMD: −1.07; 95% CI: −1.83, −0.32; *p* = 0.005; *I*^2^ = 37%; Figure 3A) and temporary (SMD: −1.34; 95% CI: −2.41, −0.27; *p* = 0.01; *I*^2^ = 46%; Figure 3A) models when compared to the control group was found. The result of the overall analysis was not changed by omitting any single study; however, subgroup analyses became non-significant after omitting data reported by Peruzzotti-Jametti et al. (2013) and Notturno et al. (2014) (Supplementary Material 4A).

Overall, the effect of cathodal tDCS was not varied by the anesthesia used during the tDCS procedure. But experiments that did not report the use of anesthesia during tDCS procedure had a negative result (SMD: −1.77; 95% CI: −3.66, 0.11; *p* = 0.06; *I*^2^ = 62%; Figure 3B). Omitting studies by Notturno et al. (2014), Zhang K. et al. (2020), and Zhang K. Y. et al. (2020) rendered the outcome of subgroup analyses non-significant (Supplementary Material 4B). However, overall analysis became significant after omitting either of the experiments by Peruzzotti-Jametti et al. (2013). Omitting the data reported by Peruzzotti-Jametti et al. (ii) resulted in a positive effect of cathodal tDCS (Supplementary Material 4B).

### Meta-Analyses on Modified Neurological Severity Score

#### The Effect of Anodal Transcranial Direct Current Stimulation on Modified Neurological Severity Score

**FIGURE 4:**
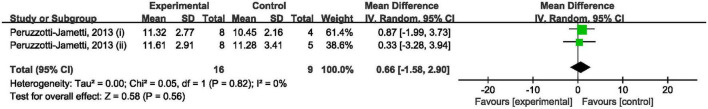
Forest plot analysis of the efficacy of anodal tDCS on mNSS compared to controls.

A study (Peruzzotti-Jametti et al., 2013) involving two independent experiments showed that anodal tDCS did not improve mNSS when compared to the control groups (SMD: 0.66; 95% CI: −1.58, 2.90; *p* = 0.56; *I*^2^ = 0%; Figure 4). Leave-one-out sensitivity analyses showed no difference in the overall finding that anodal tDCS did not improve mNSS (Supplementary Material 5).

#### The Effect of Cathodal Transcranial Direct Current Stimulation on Modified Neurological Severity Score

**FIGURE 5:**
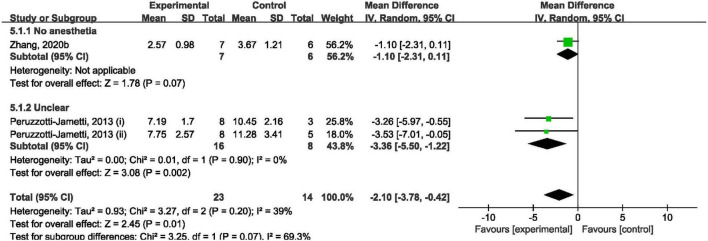
Forest plot analysis of the efficacy of cathodal tDCS on mNSS compared to controls divided on anesthesia used during tDCS procedure.

Overall, cathodal tDCS was suggested to have a positive effect by improving mNSS (Figure 5). Cathodal tDCS groups significantly improved mNSS when compared to the control groups (SMD: −2.10; 95% CI: −3.78, −0.42; *p* = 0.01; *I*^2^ = 39%). However, subgroup analysis showed that cathodal tDCS groups that did not use anesthesia during the tDCS procedure did not improve mNSS when compared to the control groups (SMD: −1.10; 95% CI: −2.31, 0.11; *p* = 0.07; Figure 5).

The analysis results became non-significant after omitting the data reported by Peruzzotti-Jametti et al. (2013) (i) (Supplementary Material 5). And overall analysis became non-significant after omitting the data reported by Peruzzotti-Jametti et al. (2013) (ii).

### Mortality and Adverse Events

Two studies (Kim et al., 2010; Peruzzotti-Jametti et al., 2013) reported mortality rates, while the remaining five studies did not report adverse events related to tDCS. Kim et al. (2010) reported that the number of dead mice was five in the anodal group, six in the cathodal group, and four in the control group. Peruzzotti-Jametti et al. (2013) reported that the overall mortality rate was 20% (6/30) in the anodal group, 6.7% (2/30) in the cathodal group, and 25% (8/32) in the control group.

## Discussion

The present study included eight studies that comprehensively evaluated the efficacy of tDCS for rodent models of ischemic stroke, and overall, our meta-analysis shows for the first time that cathodal tDCS exerts a neuroprotective effect by reducing infarct size and improving neurological deficit following focal ischemic stroke. The effect of tDCS in reducing infarct size was not varied by the duration of ischemia and anesthesia used for the intervention procedure. Cathodal tDCS without anesthesia used for the intervention procedure cannot improve neurological deficit. This review establishes a proof of concept supporting the use of cathodal tDCS as a potential paradigm for augmenting rehabilitation from ischemic stroke. However, these findings must be interpreted with caution due to the high risk of bias and the small number of studies included, resulting in only one experiment being included in some subgroup analyses.

As we know, ischemia and hypoxia may play a major role in the formation of an irreversible lesion in the core of the infarct, which cannot be rescued over 6 h following the onset (Yang et al., 2015; Zhang K. Y. et al., 2020). However, the ischemic penumbra can still be rescued if it is treated promptly and effectively (Astrup et al., 1981). Clinical investigations suggest that infarct size is linked with stroke severity (Laredo et al., 2018). Therefore, reducing infarct size has the potential to treat ischemic stroke.

Data from the present review suggest that cathodal tDCS promote the recovery of infarct size, and its effect is not varied by whether ischemia-reperfusion or not. Although reperfusion has a positive effect in some cases, ischemia-reperfusion might cause detrimental hyperemia, which is evidenced by the detrimental neuropathological outcomes and behavior observed (Olsen et al., 1981). Taken together, this shows that the positive effect of cathodal tDCS on infarct size may not be compromised by the ischemia-reperfusion injury.

There is a concern that anesthesia during intervention procedures may exert a neuroprotective effect in animal models of ischemic stroke (Archer et al., 2017), since keeping the animals awake during tDCS can avoid possible interactions between anesthetic drugs and tDCS and mimic the clinical application (Brunoni et al., 2013; Fresnoza et al., 2014). So the use of anesthesia was used to divide the subgroups. Data from the present review suggest that the effect of cathodal tDCS in reducing infarct size is not varied by the use of anesthesia. However, it is worth noting that tDCS may be combined with exercise to promote the recovery of ischemic stroke animals if animals are maintained awake. In terms of neurological deficit, cathodal tDCS without anesthesia used for the intervention procedure cannot exert a neuroprotective effect. However, no studies could be gathered to analyze the effect of cathodal tDCS under anesthesia in improving neurological deficit. As a strongly predictive of outcome 1 year following ischemic stroke, the neurological deficit should be evaluated in animal models of ischemic stroke research, to provide better evidence for clinical treatment and obtain a better outcome.

Different tDCS paradigms have been used to treat ischemic stroke, but consistent results have not been found and the mechanisms of tDCS have not yet been completely known. The tDCS parameters, such as the polarity of tDCS (anodal, cathodal, or dual) (Weinberger et al., 2017; Bastos et al., 2021), current intensity (Esmaeilpour et al., 2018), and stimulation site (Weinberger et al., 2017; Bastos et al., 2021), may cause interindividual variability in the efficacy of tDCS (Bradnam et al., 2012; Di Pino et al., 2014; Kang et al., 2016). The present meta-analysis finds evidence that cathodal tDCS is effective for reducing infarct size and improving neurological deficit. There was no evidence of improvement in infarct size and neurological deficit when anodal tDCS was used. However, it is found that both anode tDCS and cathode tDCS were significantly effective on upper limb function recovery, which is inconsistent with our conclusions in rodent models (Bai et al., 2019). One reason that must be considered and which may explain the difference between the results is that the inherent difference between meta-analysis of animal and human studies, which partly due to animal studies are so different in their species, design, and study characteristics. Another reason is that tDCS was applied as an add-on intervention in clinical trials, while tDCS was used alone in animal researches. Up to now, however, only one report about the effect of the dual tDCS in reducing infarct size and improving neurological deficit for animal models of ischemic stroke (Cheng et al., 2021). Further studies are needed to confirm the effect and mechanism of different types of tDCS on ischemic stroke to increase the likelihood of successful translation of tDCS to clinical populations.

Age and sex are critical factors for ischemic stroke (Roy-O’Reilly and McCullough, 2018). Ischemic stroke mainly occurs in elderly patients. It was reported that the crude mortality and crude incidence of stroke were both positively correlated with the proportion of the population aged ≥ 65 years (Thrift et al., 2017). However, all the included studies used young healthy animals, which is inconsistent with typical stroke patients. The difference in age may influence cerebral blood flow, angiogenesis, and neurogenesis, thus affecting the accuracy of experimental results. Therefore, age is an important factor that must be considered in preclinical studies of ischemic stroke. Besides, all the included studies solely used male animals, thus compromising the applicability of our results to females. More well-designed studies are warranted to further interpret the efficacy of tDCS for elderly and female rodents.

The timing of the application of tDCS is another important factor that must be taken into consideration. Changes related to the stage of stroke may impact the effects of tDCS. Clinical studies investigating tDCS found inconsistent results about the effect of tDCS on patients with stroke (Hesse et al., 2011; Wu et al., 2013; Viana et al., 2014). Most studies included in this meta-analysis used tDCS in an early stage of stroke, leading to the effects of tDCS in the subacute and chronic stages of stroke remain unclear. However, a meta-analysis of human studies reported that the tDCS revealed a significant effect in patients with chronic stroke rather than acute and subacute stroke, which is inconsistent with our finding obtained from rodent models (Bai et al., 2019). Of note, the meta-analysis of human studies (Bai et al., 2019) merged andol and cathodal tDCS for analysis, which may lead to different results. Furthermore, spontaneous functional recovery following a stroke occurs earlier in the rodent than in humans, making it more difficult to explore relevant neuroprotective effects of tDCS and partly resulting in different results (Schaar et al., 2010).

Besides, comorbidities are also needed to be taken into consideration in preclinical researches, as the majority of patients with stroke have suffered from comorbidities (Mergenthaler and Meisel, 2012). However, all of the included studies did not include comorbidities. Therefore, comorbidities such as diabetes, heart disease, and hypertension should be included in animal models of ischemic stroke to augment the benefit of tDCS.

The strength of this review was that it included the greatest number and most comprehensive preclinical studies to date based on the rigorous inclusion and exclusion criteria. In addition, we assessed the quality of current literature using the SYRCLE animal experiment bias risk assessment tool to increase the confidence in our results. Notwithstanding its significant findings, this study has some limitations. A limitation of this review was that there might have been several confounding factors, such as species, age, sex, stage of stroke, and diversity in the parameters of tDCS, which might have led to uncontrolled bias. Meta-analysis of animal studies is able to explore the influence of the heterogeneity, which may help in future animal research design (Hooijmans et al., 2014a; Velzen et al., 2021). An important result was that the effects of cathodal tDCS on infarct size were not influenced by ischemia duration and anesthesia. Another limitation was that the statistical power of the present meta-analysis may be restricted due to the small number of included studies and small sample sizes. Fortunately, it is recommended to pay attention to the direction of effects rather than to effect size itself in meta-analyses of animal studies, largely due to the unavoidable heterogeneity between animal researches (Hooijmans et al., 2014a). Of note, the effective endpoint of the study included was mostly the size of cerebral infarction rather than the neurological deficit assessment, which may affect the guiding significance to clinical practice. Therefore, further studies are needed to comprehensively measure the effect of tDCS on animal neurological and motor function. Lastly, all of the included studies did not indicate information such as the method of randomization and allocation concealment and some of the included studies did not report the housing condition of animals, and we thus had to estimate how these factors may affect the findings.

## Conclusion

This meta-analysis suggests that cathodal tDCS exerts a neuroprotective effect by reducing infarct size and improves neurological deficit following focal ischemic stroke. Although the findings are encouraging, the mechanisms of tDCS remain largely unknown and many fields still have not been investigated. Due to the methodological limitations and the gap between animal research and human research, it is tenuous to extrapolate the animal data to the clinical practice. There is a need for further well-designed animal studies, to explore the effect of tDCS on infarct size, neurological deficit, as well as motor function. Future animal research is needed to investigate the therapeutic mechanism and find an optimal treatment protocol in accordance with stroke therapy academic industry roundtable and stroke recovery and rehabilitation roundtable guidelines for stroke.

## Data Availability Statement

The original contributions presented in the study are included in the article/Supplementary Material, further inquiries can be directed to the corresponding author.

## Author Contributions

JH and YQ designed the study. JH performed the experiments and data analysis with the help of KZ, ZZ, and YQ. JH and KZ wrote the manuscript. All authors contributed to the article and approved the submitted version.

## Conflict of Interest

The authors declare that the research was conducted in the absence of any commercial or financial relationships that could be construed as a potential conflict of interest.

## Publisher’s Note

All claims expressed in this article are solely those of the authors and do not necessarily represent those of their affiliated organizations, or those of the publisher, the editors and the reviewers. Any product that may be evaluated in this article, or claim that may be made by its manufacturer, is not guaranteed or endorsed by the publisher.

## References

[B1] Andrade S. M. Batista L. M. Nogueira L. De Oliveira E. A. De Carvalho A. G. C. Lima S. S. (2017). Constraint-induced movement therapy combined with transcranial direct current stimulation over premotor cortex improves motor function in severe stroke: a pilot randomized controlled trial. *Rehabil. Res. Pract.* 2017:6842549. 10.1155/2017/6842549 28250992 PMC5303863

[B2] Archer D. P. Walker A. M. McCann S. K. Moser J. J. Appireddy R. M. (2017). Anesthetic neuroprotection in experimental stroke in rodents: a systematic review and meta-analysis. *Anesthesiology* 126 653–665. 10.1097/aln.0000000000001534 28182585 PMC5354268

[B3] Astrup J. Siesjö B. K. Symon L. (1981). Thresholds in cerebral ischemia - the ischemic penumbra. *Stroke* 12 723–725. 10.1161/01.str.12.6.723 6272455

[B4] Bai X. Guo Z. He L. Ren L. McClure M. A. Mu Q. (2019). Different therapeutic effects of transcranial direct current stimulation on upper and lower limb recovery of stroke patients with motor dysfunction: a meta-analysis. *Neural Plast.* 2019:1372138. 10.1155/2019/1372138 31827495 PMC6881758

[B5] Bastos R. M. de Carvalho Júnior J. G. da Silva S. A. M. Campos S. F. Rosa M. V. de Moraes Prianti B. (2021). Surgery is no more effective than conservative treatment for femoroacetabular impingement syndrome: systematic review and meta-analysis of randomized controlled trials. *Clin. Rehabil.* 35 332–341. 10.1177/0269215520966694 33143438

[B6] Beaulieu L. D. Blanchette A. K. Mercier C. Bernard-Larocque V. Milot M. H. (2019). Efficacy, safety, and tolerability of bilateral transcranial direct current stimulation combined to a resistance training program in chronic stroke survivors: a double-blind, randomized, placebo-controlled pilot study. *Restor. Neurol. Neurosci.* 37 333–346. 10.3233/rnn-190908 31227673

[B7] Bolognini N. Vallar G. Casati C. Latif L. A. El-Nazer R. Williams J. (2011). Neurophysiological and behavioral effects of tDCS combined with constraint-induced movement therapy in poststroke patients. *Neurorehabil. Neural Repair* 25 819–829. 10.1177/1545968311411056 21803933

[B8] Bradnam L. V. Stinear C. M. Barber P. A. Byblow W. D. (2012). Contralesional hemisphere control of the proximal paretic upper limb following stroke. *Cereb. Cortex* 22 2662–2671. 10.1093/cercor/bhr344 22139791 PMC4705341

[B9] Braun R. Klein R. Walter H. L. Ohren M. Freudenmacher L. Getachew K. (2016). Transcranial direct current stimulation accelerates recovery of function, induces neurogenesis and recruits oligodendrocyte precursors in a rat model of stroke. *Exp. Neurol.* 279 127–136. 10.1016/j.expneurol.2016.02.018 26923911

[B10] Brunoni A. R. Ferrucci R. Bortolomasi M. Scelzo E. Boggio P. S. Fregni F. (2013). Interactions between transcranial direct current stimulation (tDCS) and pharmacological interventions in the major depressive episode: findings from a naturalistic study. *Eur. Psychiatry* 28 356–361. 10.1016/j.eurpsy.2012.09.001 23182847

[B11] Bucur M. Papagno C. (2019). Are transcranial brain stimulation effects long-lasting in post-stroke aphasia? A comparative systematic review and meta-analysis on naming performance. *Neurosci. Biobehav. Rev.* 102 264–289. 10.1016/j.neubiorev.2019.04.019 31077693

[B12] Cheng J. Fan Y. Q. Jiang H. X. Chen S. F. Chen J. Liao X. Y. (2021). Transcranial direct-current stimulation protects against cerebral ischemia-reperfusion injury through regulating Cezanne-dependent signaling. *Exp. Neurol.* 345:113818. 10.1016/j.expneurol.2021.113818 34324860

[B13] Di Pino G. Pellegrino G. Assenza G. Capone F. Ferreri F. Formica D. (2014). Modulation of brain plasticity in stroke: a novel model for neurorehabilitation. *Nat. Rev. Neurol.* 10 597–608. 10.1038/nrneurol.2014.162 25201238

[B14] Esmaeilpour Z. Marangolo P. Hampstead B. M. Bestmann S. Galletta E. Knotkova H. (2018). Incomplete evidence that increasing current intensity of tDCS boosts outcomes. *Brain Stimul.* 11 310–321. 10.1016/j.brs.2017.12.002 29258808 PMC7050474

[B15] Fan J. Li Y. Yang Y. Qu Y. Li S. (2017). Efficacy of noninvasive brain stimulation on unilateral neglect after stroke: a systematic review and meta-analysis. *Am. J. Phys. Med. Rehabil.* 97 261–269. 10.1097/phm.0000000000000834 28953034

[B16] Fresnoza S. Stiksrud E. Klinker F. (2014). Dosage-dependent effect of dopamine D2 receptor activation on motor cortex plasticity in humans. *J. Neurosci.* 34 10701–10709. 10.1523/jneurosci.0832-14.2014 25100602 PMC4122803

[B17] Fritsch B. Reis J. Martinowich K. Schambra H. M. Ji Y. Cohen L. G. (2010). Direct current stimulation promotes BDNF-dependent synaptic plasticity: potential implications for motor learning. *Neuron* 66 198–204. 10.1016/j.neuron.2010.03.035 20434997 PMC2864780

[B18] Guan T. Ma J. Li M. Xue T. Lan Z. Guo J. (2017). Rapid transitions in the epidemiology of stroke and its risk factors in China from 2002 to 2013. *Neurology* 89 53–61. 10.1212/WNL.0000000000004056 28566547

[B19] Hesse S. Waldner A. Mehrholz J. Tomelleri C. Pohl M. Werner C. (2011). Combined transcranial direct current stimulation and robot-assisted arm training in subacute stroke patients: an exploratory, randomized multicenter trial. *Neurorehabil. Neural Repair* 25 838–846. 10.1177/1545968311413906 21825004

[B20] Hooijmans C. R. IntHout J. Ritskes-Hoitinga M. Rovers M. M. (2014a). Meta-analyses of animal studies: an introduction of a valuable instrument to further improve healthcare. *ILAR J.* 55 418–426. 10.1093/ilar/ilu042 25541544 PMC4276598

[B21] Hooijmans C. R. Rovers M. M. de Vries R. B. Leenaars M. Ritskes-Hoitinga M. Langendam M. W. (2014b). SYRCLE’s risk of bias tool for animal studies. *BMC Med. Res. Methodol.* 14:43. 10.1186/1471-2288-14-43 24667063 PMC4230647

[B22] Johnson C. A. Burridge J. H. Strike P. W. Wood D. E. Swain I. D. (2004). The effect of combined use of botulinum toxin type A and functional electric stimulation in the treatment of spastic drop foot after stroke: a preliminary investigation. *Arch. Gerontol. Geriatr.* 85 902–909. 10.1016/j.apmr.2003.08.081 15179643

[B23] Kang N. Summers J. J. Cauraugh J. H. (2016). Transcranial direct current stimulation facilitates motor learning post-stroke: a systematic review and meta-analysis. *J. Neurol. Neurosurg. Psychiatry* 87 345–355. 10.1136/jnnp-2015-311242 26319437

[B24] Kim S. J. Bang M. S. Han T. R. Kim B. K. Ko Y. J. Kim M. H. (2010). Functional and histologic changes after repeated transcranial direct current stimulation in rat stroke model. *J. Korean Med. Sci.* 25 1499–1505. 10.3346/jkms.2010.25.10.1499 20890433 PMC2946662

[B25] Laredo C. Zhao Y. Rudilosso S. Renú A. Pariente J. C. Chamorro Á (2018). Prognostic significance of infarct size and location: the case of insular stroke. *Sci. Rep.* 8:9498. 10.1038/s41598-018-27883-3 29934530 PMC6015086

[B26] Liebetanz D. Nitsche M. A. Tergau F. Paulus W. (2002). Pharmacological approach to the mechanisms of transcranial DC-stimulation-induced after-effects of human motor cortex excitability. *Brain* 125(Pt. 10), 2238–2247. 10.1093/brain/awf238 12244081

[B27] Machado D. Unal G. Andrade S. M. Moreira A. Altimari L. R. Brunoni A. R. (2019). Effect of transcranial direct current stimulation on exercise performance: a systematic review and meta-analysis. *Brain Stimul.* 12 593–605. 10.1016/j.brs.2018.12.227 30630690

[B28] Mergenthaler P. Meisel A. (2012). Do stroke models model stroke? *Dis. Model. Mech.* 5 718–725. 10.1242/dmm.010033 23115201 PMC3484854

[B29] Moretti A. Ferrari F. Villa R. F. (2015). Neuroprotection for ischaemic stroke: current status and challenges. *Pharmacol. Ther*. 146, 23–34. 10.1016/j.pharmthera.2014.09.003 25196155

[B30] Nitsche M. A. Paulus W. (2001). Sustained excitability elevations induced by transcranial DC motor cortex stimulation in humans. *Neurology* 57 1899–1901. 10.1212/wnl.57.10.1899 11723286

[B31] Nitsche M. A. Fricke K. Henschke U. Schlitterlau A. Liebetanz D. Lang N. (2003a). Pharmacological modulation of cortical excitability shifts induced by transcranial direct current stimulation in humans. *J. Physiol.* 553(Pt. 1), 293–301. 10.1113/jphysiol.2003.049916 12949224 PMC2343495

[B32] Nitsche M. A. Liebetanz D. Antal A. Lang N. Tergau F. Paulus W. (2003b). Modulation of cortical excitability by weak direct current stimulation–technical, safety and functional aspects. *Suppl. Clin. Neurophysiol.* 56 255–276. 10.1016/s1567-424x(09)70230-2 14677403

[B33] Notturno F. Pace M. Uncini A. Zappasodi F. Cam E. Bassetti C. L. (2014). Neuroprotective effect of cathodal transcranial direct current stimulation in a rat stroke model. *J. Neurol. Sci.* 342 146–151. 10.1016/j.jns.2014.05.017 24857352

[B34] Olsen T. S. Larsen B. Skriver E. B. Herning M. Enevoldsen E. Lassen N. A. (1981). Focal cerebral hyperemia in acute stroke. incidence, pathophysiology and clinical significance. *Stroke* 12 598–607. 10.1161/01.str.12.5.598 6975512

[B35] Peruzzotti-Jametti L. Bacigaluppi M. Gallizioli M. Sandrone S. Martino G. Cambiaghi M. (2013). Safety and efficacy of transcranial direct current stimulation in acute experimental ischemic stroke. *Stroke* 44 3166–3174. 10.1161/STROKEAHA.113.001687 23982710

[B36] Qu Y. P. Wu D. Y. Tu X. Q. Qian L. Yang Y. B. Geng H. (2009). Effects of transcranial direct current stimulation on relieving upper-limb spasticity after stroke (in Chinese). *Chin. J. Cerebrovasc.* 6 586–589.

[B37] Rocha S. Silva E. Foerster Á Wiesiolek C. Chagas A. P. Machado G. (2016). The impact of transcranial direct current stimulation (tDCS) combined with modified constraint-induced movement therapy (mCIMT) on upper limb function in chronic stroke: a double-blind randomized controlled trial. *Disabil. Rehabil.* 38 653–660. 10.3109/09638288.2015.1055382 26061222

[B38] Roy-O’Reilly M. McCullough L. D. (2018). Age and sex are critical factors in ischemic stroke pathology. *Endocrinology* 159 3120–3131. 10.1210/en.2018-00465 30010821 PMC6963709

[B39] Schaar K. L. Brenneman M. M. Savitz S. I. (2010). Functional assessments in the rodent stroke model. *Exp. Transl. Stroke Med.* 2:13. 10.1186/2040-7378-2-13 20642841 PMC2915950

[B40] Takebayashi T. Takahashi K. Moriwaki M. Sakamoto T. Domen K. (2017). Improvement of upper extremity deficit after constraint-induced movement therapy combined with and without preconditioning stimulation using dual-hemisphere transcranial direct current stimulation and peripheral neuromuscular stimulation in chronic stroke patients: a pilot randomized controlled trial. *Front. Neurol.* 8:568. 10.3389/fneur.2017.00568 29163334 PMC5670104

[B41] Thrift A. G. Thayabaranathan T. Howard G. Howard V. J. Rothwell P. M. Feigin V. L. (2017). Global stroke statistics. *Int. J. Stroke* 12 13–32. 10.1177/1747493016676285 27794138

[B42] Velzen M. V. Dahan J. D. C. van Dorp E. L. A. Mogil J. S. Hooijmans C. R. Dahan A. (2021). Efficacy of ketamine in relieving neuropathic pain: a systematic review and meta-analysis of animal studies. *Pain* 162 2320–2330. 10.1097/j.pain.0000000000002231 33790195 PMC8374709

[B43] Viana R. T. Laurentino G. E. C. Souza R. J. P. Fonseca J. B. Silva Filho E. M. Dias S. N. (2014). Effects of the addition of transcranial direct current stimulation to virtual reality therapy after stroke: a pilot randomized controlled trial. *NeuroRehabilitation* 34 437–446. 10.3233/NRE-141065 24473248

[B44] Weinberger A. B. Green A. E. Chrysikou E. G. (2017). Using transcranial direct current stimulation to enhance creative cognition: interactions between task, polarity, and stimulation site. *Front. Hum. Neurosci.* 11:246. 10.3389/fnhum.2017.00246 28559804 PMC5432551

[B45] Wu D. Qian L. Zorowitz R. D. Zhang L. Qu Y. Yuan Y. (2013). Effects on decreasing upper-limb poststroke muscle tone using transcranial direct current stimulation: a randomized sham-controlled study. *Arch. Phys. Med. Rehabil.* 94 1–8. 10.1016/j.apmr.2012.07.022 22878231

[B46] Wu S. Wu B. Liu M. Chen Z. Wang W. Anderson C. S. (2019). Stroke in China: advances and challenges in epidemiology, prevention, and management. *Lancet Neurol.* 18 394–405. 10.1016/S1474-4422(18)30500-3 30878104

[B47] Yang Y. Sandhu H. K. Zhi F. Hua F. Wu M. Xia Y. (2015). Effects of hypoxia and ischemia on microRNAs in the brain. *Curr. Med. Chem.* 22 1292–1301. 10.2174/0929867322666150209154755 25666793

[B48] Yoon K. J. Oh B.-M. Kim D.-Y. (2012). Functional improvement and neuroplastic effects of anodal transcranial direct current stimulation (tDCS) delivered 1 day vs. 1 week after cerebral ischemia in rats. *Brain Res.* 1452 61–72. 10.1016/j.brainres.2012.02.062 22444278

[B49] Zhang K. Y. Rui G. Zhang J. P. Guo L. An G. Z. Lin J. J. (2020). Cathodal tDCS exerts neuroprotective effect in rat brain after acute ischemic stroke. *BMC Neurosci.* 21:21. 10.1186/s12868-020-00570-8 32397959 PMC7216334

[B50] Zhang K. Guo L. Zhang J. Rui G. An G. Zhou Y. (2020). tDCS accelerates the rehabilitation of MCAO-induced motor function deficits *via* neurogenesis modulated by the notch1 signaling pathway. *Neurorehabil. Neural Repair* 34 640–651. 10.1177/1545968320925474 32543269

