# Supplementary material for: Neuroprotection by Transcranial Direct Current Stimulation in Rodent Models of Focal Ischemic Stroke: A Meta-Analysis
Source: Front Neurosci. 2021 Nov 23;15:761971. doi: 10.3389/fnins.2021.761971 (PMC8649802; doi:10.3389/fnins.2021.761971)
Supplement: Supplementary file 1 [file Table_1.DOCX]

**Supplementary material**

**Supplementary 1:** PRISMA checklist

**Supplementary 2:** Literature search strategy used in the current review.

**Supplementary 3:** Quality assessment of included studies.

**Supplementary 4:** Results of leave-one-out sensitivity analysis investigating the effect of tDCS on infarct size.

**Supplementary 5:** Results of leave-one-out sensitivity analysis investigating the effect of tDCS on mNSS.

**Supplement 1.** PRISMA checklist.

| **Section/topic** | **#** | **Checklist item** | **Reported on page #** |
| --- | --- | --- | --- |
| **TITLE** | | |  |
| Title | 1 | Identify the report as a systematic review, meta-analysis, or both. | 1 |
| **ABSTRACT** | | |  |
| Structured summary | 2 | Provide a structured summary including, as applicable: background; objectives; data sources; study eligibility criteria, participants, and interventions; study appraisal and synthesis methods; results; limitations; conclusions and implications of key findings; systematic review registration number. | 1 |
| **INTRODUCTION** | | |  |
| Rationale | 3 | Describe the rationale for the review in the context of what is already known. | 2 |
| Objectives | 4 | Provide an explicit statement of questions being addressed with reference to participants, interventions, comparisons, outcomes, and study design (PICOS). | 2 |
| **METHODS** | | |  |
| Protocol and registration | 5 | Indicate if a review protocol exists, if and where it can be accessed (e.g., Web address), and, if available, provide registration information including registration number. | 2 |
| Eligibility criteria | 6 | Specify study characteristics (e.g., PICOS, length of follow-up) and report characteristics (e.g., years considered, language, publication status) used as criteria for eligibility, giving rationale. | 3 |
| Information sources | 7 | Describe all information sources (e.g., databases with dates of coverage, contact with study authors to identify additional studies) in the search and date last searched. | 3 |
| Search | 8 | Present full electronic search strategy for at least one database, including any limits used, such that it could be repeated. | S2 |
| Study selection | 9 | State the process for selecting studies (i.e., screening, eligibility, included in systematic review, and, if applicable, included in the meta-analysis). | 3 |
| Data collection process | 10 | Describe method of data extraction from reports (e.g., piloted forms, independently, in duplicate) and any processes for obtaining and confirming data from investigators. | 3 |
| Data items | 11 | List and define all variables for which data were sought (e.g., PICOS, funding sources) and any assumptions and simplifications made. | 3 |
| Risk of bias in individual studies | 12 | Describe methods used for assessing risk of bias of individual studies (including specification of whether this was done at the study or outcome level), and how this information is to be used in any data synthesis. | 3 |
| Summary measures | 13 | State the principal summary measures (e.g., risk ratio, difference in means). | 3 |
| Synthesis of results | 14 | Describe the methods of handling data and combining results of studies, if done, including measures of consistency (e.g., I^2^) for each meta-analysis. | 3 |
| Risk of bias across studies | 15 | Specify any assessment of risk of bias that may affect the cumulative evidence (e.g., publication bias, selective reporting within studies). | 3 |
| Additional analyses | 16 | Describe methods of additional analyses (e.g., sensitivity or subgroup analyses, meta-regression), if done, indicating which were pre-specified. | 3 |
| **RESULTS** | | |  |
| Study selection | 17 | Give numbers of studies screened, assessed for eligibility, and included in the review, with reasons for exclusions at each stage, ideally with a flow diagram. | 4  Figure 1 |
| Study characteristics | 18 | For each study, present characteristics for which data were extracted (e.g., study size, PICOS, follow-up period) and provide the citations. | 4  Table 1-2 |
| Risk of bias within studies | 19 | Present data on risk of bias of each study and, if available, any outcome level assessment (see item 12). | 4  S3 |
| Results of individual studies | 20 | For all outcomes considered (benefits or harms), present, for each study: (a) simple summary data for each intervention group (b) effect estimates and confidence intervals, ideally with a forest plot. | Figure 2-5 |
| Synthesis of results | 21 | Present results of each meta-analysis done, including confidence intervals and measures of consistency. | 5-6  Figure 2-5 |
| Risk of bias across studies | 22 | Present results of any assessment of risk of bias across studies (see Item 15). | 4-5  S3 |
| Additional analysis | 23 | Give results of additional analyses, if done (e.g., sensitivity or subgroup analyses, meta-regression [see Item 16]). | 5-6  Figure 2-5 |
| **DISCUSSION** | | |  |
| Summary of evidence | 24 | Summarize the main findings including the strength of evidence for each main outcome; consider their relevance to key groups (e.g., healthcare providers, users, and policy makers). | 6 |
| Limitations | 25 | Discuss limitations at study and outcome level (e.g., risk of bias), and at review-level (e.g., incomplete retrieval of identified research, reporting bias). | 7-8 |
| Conclusions | 26 | Provide a general interpretation of the results in the context of other evidence, and implications for future research. | 8 |
| **FUNDING** | | |  |
| Funding | 27 | Describe sources of funding for the systematic review and other support (e.g., supply of data); role of funders for the systematic review. | 8 |

**Supplement 2.** Literature search strategy used in the current review.

Database: MEDLINE (via PubMed, to August 2021)

- #1 (stroke[MeSH Terms]) OR (stroke*[Title/Abstract] OR apoplex* [Title/Abstract] OR "brain vascular accident"[Title/Abstract] OR "brain vascular accidents"[Title/Abstract] OR cva*[Title/Abstract] OR "cerebrovascular accident"[Title/Abstract] OR "cerebrovascular accidents"[Title/Abstract] OR "brain ischemia"[Title/Abstract] OR "brain ischemias"[Title/Abstract] OR "cerebral ischemia"[Title/Abstract] OR "cerebral ischemias"[Title/Abstract] OR "ischemic encephalopathies"[Title/Abstract] OR "ischemic encephalopathy"[Title/Abstract] OR "anterior choroidal artery infarction"[Title/Abstract] OR "cerebral infarction"[Title/Abstract] OR "cerebral infarctions"[Title/Abstract] OR "posterior choroidal artery infarction"[Title/Abstract] OR "subcortical infarction"[Title/Abstract] OR "subcortical infarctions"[Title/Abstract] OR "anoxic ischemic encephalopathy"[Title/Abstract] OR "anoxic-ischemic encephalopathies"[Title/Abstract] OR "anoxic-ischemic encephalopathy"[Title/Abstract] OR "brain anoxia ischemia"[Title/Abstract] OR "brain anoxia-ischemia"[Title/Abstract] OR "brain anoxia-ischemias"[Title/Abstract] OR "brain hypoxia ischemia"[Title/Abstract] OR "brain hypoxia-ischemia"[Title/Abstract] OR "brain hypoxia-ischemias"[Title/Abstract] OR "brain ischemia anoxia"[Title/Abstract] OR "brain ischemia hypoxia"[Title/Abstract] OR "brain ischemia-anoxia"[Title/Abstract] OR "brain ischemia-anoxias"[Title/Abstract] OR "brain ischemia-hypoxia"[Title/Abstract] OR "brain ischemia-hypoxias"[Title/Abstract] OR "cerebral anoxia ischemia"[Title/Abstract] OR "cerebral anoxia-ischemia"[Title/Abstract] OR "cerebral anoxia-ischemias"[Title/Abstract] OR "cerebral hypoxia ischemia" [Title/Abstract] OR "cerebral hypoxia-ischemia"[Title/Abstract] OR "cerebral hypoxia-ischemias"[Title/Abstract] OR "cerebral ischemia anoxia"[Title/Abstract] OR "cerebral ischemia hypoxia"[Title/Abstract] OR "cerebral ischemia-anoxia"[Title/Abstract] OR "cerebral ischemia-anoxias"[Title/Abstract] OR "cerebral ischemia-hypoxia"[Title/Abstract] OR "cerebral ischemia-hypoxias"[Title/Abstract] OR "hypoxic ischemic encephalopathy"[Title/Abstract] OR "hypoxic-ischemic encephalopathies"[Title/Abstract] OR "hypoxic- ischemic encephalopathy"[Title/Abstract] OR "ischemic hypoxic encephalopathy"[Title/Abstract] OR "ischemic-hypoxic encephalopathies"[Title/Abstract] OR "ischemic-hypoxic encephalopathy"[Title/Abstract])
- #2 (Transcranial Direct Current Stimulation[MeSH Terms]) OR (''Transcranial Direct Current Stimulation''[Title/Abstract] OR tDCS[Title/Abstract] OR tDCSs[Title/Abstract] OR ''Transcranial Electrical Stimulation''[Title/Abstract] OR ''Electrical Stimulation, Transcranial''[Title/Abstract] OR ''Electrical Stimulations, Transcranial''[Title/Abstract] OR ''Stimulation, Transcranial Electrical''[Title/Abstract] OR ''Stimulations, Transcranial Electrical''[Title/Abstract] OR ''Transcranial Electrical Stimulations''[Title/Abstract])
- #3 (Muridae[MeSH Terms]) OR (Muridae[Title/Abstract] OR mouse[Title/Abstract] OR mice[Title/Abstract] OR rat[Title/Abstract] OR rats[Title/Abstract] OR rodent*[Title/Abstract] OR murine[Title/Abstract])
- #4 #1 AND #2 AND #3

Database: EMBASE (to August 2021)

- #1 exp stroke/
- #2 (stroke* OR apoplex* OR "brain vascular accident" OR "brain vascular accidents" OR cva* OR "cerebrovascular accident" OR "cerebrovascular accidents" OR "brain ischemia" OR "brain ischemias" OR "cerebral ischemia" OR "cerebral ischemias" OR "ischemic encephalopathies" OR "ischemic encephalopathy" OR "anterior choroidal artery infarction" OR "cerebral infarction" OR "cerebral infarctions" OR "posterior choroidal artery infarction" OR "subcortical infarction" OR "subcortical infarctions" OR "anoxic ischemic encephalopathy" OR "anoxic-ischemic encephalopathies" OR "anoxic-ischemic encephalopathy" OR "brain anoxia ischemia" OR "brain anoxia-ischemia" OR "brain anoxia-ischemias" OR "brain hypoxia ischemia" OR "brain hypoxia-ischemia" OR "brain hypoxia-ischemias" OR "brain ischemia anoxia" OR "brain ischemia hypoxia" OR "brain ischemia-anoxia" OR "brain ischemia-anoxias" OR "brain ischemia-hypoxia" OR "brain ischemia-hypoxias" OR "cerebral anoxia ischemia" OR "cerebral anoxia-ischemia" OR "cerebral anoxia-ischemias" OR "cerebral hypoxia ischemia" OR "cerebral hypoxia-ischemia" OR "cerebral hypoxia-ischemias" OR "cerebral ischemia anoxia" OR "cerebral ischemia hypoxia" OR "cerebral ischemia-anoxia" OR "cerebral ischemia-anoxias" OR "cerebral ischemia-hypoxia" OR "cerebral ischemia-hypoxias" OR "hypoxic ischemic encephalopathy" OR "hypoxic-ischemic encephalopathies" OR "hypoxic-ischemic encephalopathy" OR "ischemic hypoxic encephalopathy" OR "ischemic-hypoxic encephalopathies" OR "ischemic-hypoxic encephalopathy").ab.ti.
- #3 1 or 2
- #4 exp Transcranial Direct Current Stimulation/
- #5 (''Transcranial Direct Current Stimulation'' OR tDCS OR tDCSs OR ''Transcranial Electrical Stimulation'' OR ''Electrical Stimulation, Transcranial'' OR ''Electrical Stimulations, Transcranial'' OR ''Stimulation, Transcranial Electrical'' OR ''Stimulations, Transcranial Electrical'' OR ''Transcranial Electrical Stimulations'').ab.ti.
- #6 4 or 5
- #7 exp Muridae/
- #8 (Muridae OR mouse OR mice OR rat OR rats OR rodent* OR murine).ab,ti.
- #9 7 or 8
- #9 3 and 6 and 9

Database: Web of science (to August 2021)

- #1 TS=stroke
- # 2 TI=(stroke* OR apoplex* OR "brain vascular accident" OR "brain vascular accidents" OR cva* OR "cerebrovascular accident" OR "cerebrovascular accidents" OR "brain ischemia" OR "brain ischemias" OR "cerebral ischemia" OR "cerebral ischemias" OR "ischemic encephalopathies" OR "ischemic encephalopathy" OR "anterior choroidal artery infarction" OR "cerebral infarction" OR "cerebral infarctions" OR "posterior choroidal artery infarction" OR "subcortical infarction" OR "subcortical infarctions" OR "anoxic ischemic encephalopathy" OR "anoxic-ischemic encephalopathies" OR "anoxic-ischemic encephalopathy" OR "brain anoxia ischemia" OR "brain anoxia-ischemia" OR "brain anoxia-ischemias" OR "brain hypoxia ischemia" OR "brain hypoxia-ischemia" OR "brain hypoxia-ischemias" OR "brain ischemia anoxia" OR "brain ischemia hypoxia" OR "brain ischemia-anoxia" OR "brain ischemia-anoxias" OR "brain ischemia-hypoxia" OR "brain ischemia-hypoxias" OR "cerebral anoxia ischemia" OR "cerebral anoxia-ischemia" OR "cerebral anoxia-ischemias" OR "cerebral hypoxia ischemia" OR "cerebral hypoxia-ischemia" OR "cerebral hypoxia-ischemias" OR "cerebral ischemia anoxia" OR "cerebral ischemia hypoxia" OR "cerebral ischemia-anoxia" OR "cerebral ischemia-anoxias" OR "cerebral ischemia-hypoxia" OR "cerebral ischemia-hypoxias" OR "hypoxic ischemic encephalopathy" OR "hypoxic-ischemic encephalopathies" OR "hypoxic-ischemic encephalopathy" OR "ischemic hypoxic encephalopathy" OR "ischemic-hypoxic encephalopathies" OR "ischemic-hypoxic encephalopathy") OR KP=(stroke* OR apoplex* OR "brain vascular accident" OR "brain vascular accidents" OR cva* OR "cerebrovascular accident" OR "cerebrovascular accidents" OR "brain ischemia" OR "brain ischemias" OR "cerebral ischemia" OR "cerebral ischemias" OR "ischemic encephalopathies" OR "ischemic encephalopathy" OR "anterior choroidal artery infarction" OR "cerebral infarction" OR "cerebral infarctions" OR "posterior choroidal artery infarction" OR "subcortical infarction" OR "subcortical infarctions" OR "anoxic ischemic encephalopathy" OR "anoxic-ischemic encephalopathies" OR "anoxic-ischemic encephalopathy" OR "brain anoxia ischemia" OR "brain anoxia-ischemia" OR "brain anoxia-ischemias" OR "brain hypoxia ischemia" OR "brain hypoxia-ischemia" OR "brain hypoxia-ischemias" OR "brain ischemia anoxia" OR "brain ischemia hypoxia" OR "brain ischemia-anoxia" OR "brain ischemia-anoxias" OR "brain ischemia-hypoxia" OR "brain ischemia-hypoxias" OR "cerebral anoxia ischemia" OR "cerebral anoxia-ischemia" OR "cerebral anoxia-ischemias" OR "cerebral hypoxia ischemia" OR "cerebral hypoxia-ischemia" OR "cerebral hypoxia-ischemias" OR "cerebral ischemia anoxia" OR "cerebral ischemia hypoxia" OR "cerebral ischemia-anoxia" OR "cerebral ischemia-anoxias" OR "cerebral ischemia-hypoxia" OR "cerebral ischemia-hypoxias" OR "hypoxic ischemic encephalopathy" OR "hypoxic-ischemic encephalopathies" OR "hypoxic-ischemic encephalopathy" OR "ischemic hypoxic encephalopathy" OR "ischemic-hypoxic encephalopathies" OR "ischemic-hypoxic encephalopathy")
- # 3 #2 OR #1
- # 4 TS=Transcranial Direct Current Stimulation
- # 5 TI=(''Transcranial Direct Current Stimulation'' OR tDCS OR tDCSs OR ''Transcranial Electrical Stimulation'' OR ''Electrical Stimulation, Transcranial'' OR ''Electrical Stimulations, Transcranial'' OR ''Stimulation, Transcranial Electrical'' OR ''Stimulations, Transcranial Electrical'' OR ''Transcranial Electrical Stimulations'') OR KP=(''Transcranial Direct Current Stimulation'' OR tDCS OR tDCSs OR ''Transcranial Electrical Stimulation'' OR ''Electrical Stimulation, Transcranial'' OR ''Electrical Stimulations, Transcranial'' OR ''Stimulation, Transcranial Electrical'' OR ''Stimulations, Transcranial Electrical'' OR ''Transcranial Electrical Stimulations'')
- # 6 #5 OR #4
- # 7 TS=Muridae
- # 8 TI=(Muridae OR mouse OR mice OR rat OR rats OR rodent* OR murine) OR KP=(Muridae OR mouse OR mice OR rat OR rats OR rodent* OR murine)
- #9 #7 OR #8
- #10 #9 AND #6 AND #3

Database: SCOPUS (to August 2021)

TITLE-ABS-KEY ( stroke* OR apoplex* OR "brain vascular accident" OR "brain vascular accidents" OR cva* OR "cerebrovascular accident" OR "cerebrovascular accidents" OR "brain ischemia" OR "brain ischemias" OR "cerebral ischemia" OR "cerebral ischemias" OR "ischemic encephalopathies" OR "ischemic encephalopathy" OR "anterior choroidal artery infarction" OR "cerebral infarction" OR "cerebral infarctions" OR "posterior choroidal artery infarction" OR "subcortical infarction" OR "subcortical infarctions" OR "anoxic ischemic encephalopathy" OR "anoxic-ischemic encephalopathies" OR "anoxic-ischemic encephalopathy" OR "brain anoxia ischemia" OR "brain anoxia-ischemia" OR "brain anoxia-ischemias" OR "brain hypoxia ischemia" OR "brain hypoxia-ischemia" OR "brain hypoxia-ischemias" OR "brain ischemia anoxia" OR "brain ischemia hypoxia" OR "brain ischemia-anoxia" OR "brain ischemia-anoxias" OR "brain ischemia-hypoxia" OR "brain ischemia-hypoxias" OR "cerebral anoxia ischemia" OR "cerebral anoxia-ischemia" OR "cerebral anoxia-ischemias" OR "cerebral hypoxia ischemia" OR "cerebral hypoxia-ischemia" OR "cerebral hypoxia-ischemias" OR "cerebral ischemia anoxia" OR "cerebral ischemia hypoxia" OR "cerebral ischemia-anoxia" OR "cerebral ischemia-anoxias" OR "cerebral ischemia-hypoxia" OR "cerebral ischemia-hypoxias" OR "hypoxic ischemic encephalopathy" OR "hypoxic-ischemic encephalopathies" OR "hypoxic-ischemic encephalopathy" OR "ischemic hypoxic encephalopathy" OR "ischemic-hypoxic encephalopathies" OR "ischemic-hypoxic encephalopathy" ) AND TITLE-ABS-KEY ( ''transcranial AND direct AND current AND stimulation'' OR tdcs OR tdcss OR ''transcranial AND electrical AND stimulation'' OR ''electrical AND stimulation, AND transcranial'' OR ''electrical AND stimulations, AND transcranial'' OR ''stimulation, AND transcranial AND electrical'' OR ''stimulations, AND transcranial AND electrical'' OR ''transcranial AND electrical AND stimulations'' ) AND TITLE-ABS-KEY ( muridae OR mouse OR mice OR rat OR rats OR rodent* OR murine )

**Supplement 3.** Quality assessment of included studies.

| Item | Bias | Braun 2016 | Kim 2010 | Notturno 2013 | Peruzzotti-Jametti 2013 | Yoon 2012 | Zhang 2020a | Zhang 2020b | Cheng 2021 |
| --- | --- | --- | --- | --- | --- | --- | --- | --- | --- |
| 1 | Sequence generation (Selection bias) | U | U | U | U | U | U | U | U |
| 2 | Baseline characteristics (Selection bias) | U | Y | U | Y | Y | Y | Y | U |
| 3 | Allocation concealment (Selection bias) | U | U | U | U | U | U | U | U |
| 4 | Random housing (Performance bias) | U | U | Y | U | Y | Y | Y | Y |
| 5 | Blinding (Performance bias) | U | U | U | Y | U | U | U | Y |
| 6 | Random outcome assessment (Detection bias) | U | U | U | Y | U | U | U | Y |
| 7 | Blinding (Detection bias) | Y | Y | U | Y | Y | Y | Y | Y |
| 8 | Incomplete outcome data (Attrition bias) | N | N | U | U | U | U | U | U |
| 9 | Selective outcome reporting (Reporting bias) | Y | Y | Y | Y | Y | Y | Y | Y |
| 10 | Other sources of bias | U | U | U | U | U | U | U | U |

Y: low risk of bias; N, high risk of bias; U, unclear risk of bias.

**Supplement 4.** Results of leave-one-out sensitivity analysis investigating the effect of tDCS on infarct size.

1. Leave one out sensitivity analysis when grouping studies according to the duration of ischemia (Figure 2a)

|  |  |  | Subgroup analysis | | | Overall analysis | | |
| --- | --- | --- | --- | --- | --- | --- | --- | --- |
| Polarity | Subgroup | Study excluded | *I*^2^ (%) | SMD | *p* | *I*^2^ (%) | SMD | *p* |
| Anodal | Permanent ischemia | - | - | - | - | - | - | - |
|  | Temporary ischemia | Braun | 55 | -0.11 [-0.98, 0.77] | 0.81 | 45 | -0.02 [-0.77, 0.72] | 0.96 |
|  |  | Peruzzotti-Jametti (i) | 0 | -0.43 [-0.98, 0.13] | 0.13 | 0 | -0.32 [-0.84, 0.20] | 0.23 |
|  |  | Peruzzotti-Jametti (ii) | 55 | -0.11 [-0.97, 0.76] | 0.81 | 45 | -0.02 [-0.76, 0.71] | 0.95 |
|  |  | Yong (early) | 45 | 0.02 [-0.76, 0.81] | 0.96 | 32 | 0.08 [-0.58, 0.74] | 0.82 |
|  |  | Yong (late) | 48 | 0.01 [-0.80, 0.81] | 0.99 | 35 | 0.07 [-0.61, 0.75] | 0.84 |
| Cathodal | Permanent ischemia | Kim | 0 | -1.38 [-2.02, -0.74] | *<*0.0001 | 23 | -1.27 [-1.85, -0.69] | *<*0.0001 |
|  |  | Notturno (i) | 60 | -0.77 [-2.06, 0.51] | 0.24 | 40 | -1.07 [-1.80, -0.35] | 0.004 |
|  |  | Notturno (ii) | 65 | -0.82 [-2.21, 0.58] | 0.25 | 42 | -1.10 [-1.84, -0.36] | 0.003 |
|  | Temporary ischemia | Braun | 9 | -1.70 [-2.69, -0.71] | 0.0007 | 18 | -1.29 [-1.86, -0.73] | *<*0.0001 |
|  |  | Peruzzotti-Jametti (i) | 18 | -0.87 [-1.77, 0.03] | 0.06 | 18 | -1.00 [-1.53, -0.46] | 0.0002 |
|  |  | Peruzzotti-Jametti (ii) | 59 | -1.65 [-3.25, -0.05] | 0.04 | 43 | -1.17 [-1.88, -0.46] | 0.001 |
|  |  | Zhang* | 57 | -1.33 [-2.61, -0.05] | 0.04 | 41 | -1.09 [-1.74, -0.45] | 0.0008 |
|  |  | Zhang | 51 | -1.19 [-2.32, -0.07] | 0.04 | 36 | -1.07 [-1.67, -0.47] | 0.0005 |

1. Leave one out sensitivity analysis when grouping studies according to the anesthesia used during tDCS procedures (Figure 2b)

|  |  |  | Subgroup analysis | | | Overall analysis | | |
| --- | --- | --- | --- | --- | --- | --- | --- | --- |
| Polarity | Subgroup | Study excluded | *I*^2^ (%) | SMD | *p* | *I*^2^ (%) | SMD | *p* |
| Anodal | Anesthesia | Braun | 0 | -0.44 [-1.14, 0.26] | 0.22 | 45 | -0.02 [-0.77, 0.72] | 0.95 |
|  |  | Kim | 0 | -0.51 [-1.14, 0.13] | 0.12 | 40 | -0.14 [-0.80, 0.52] | 0.68 |
|  |  | Yong (early) | 0 | -0.22 [-0.92, 0.47] | 0.52 | 32 | 0.08 [-0.58, 0.74] | 0.82 |
|  |  | Yong (late) | 0 | -0.25 [-0.94, 0.44] | 0.48 | 35 | 0.07 [-0.61, 0.75] | 0.84 |
|  | Unclear | Peruzzotti-Jametti (i) | - | -0.18 [-1.30, 0.94] | 0.76 | 0 | -0.32 [-0.84, 0.20] | 0.23 |
|  |  | Peruzzotti-Jametti (ii) | - | 1.40 [0.02, 2.79] | 0.05 | 45 | -0.02 [-0.76, 0.71] | 0.95 |
| Cathodal | Anesthesia | Braun | 37 | -1.07 [-1.83, -0.32] | 0.005 | 18 | -1.29 [-1.86, -0.73] | 0.0001 |
|  |  | Kim | 43 | -1.04 [-1.79, -0.29] | 0.007 | 23 | -1.27 [-1.85, -0.69] | *<*0.0001 |
|  |  | Notturno (i) | 48 | -0.58 [-1.49, 0.33] | 0.21 | 40 | -1.07 [-1.80, -0.35] | 0.004 |
|  |  | Notturno (ii) | 55 | -0.61 [-1.59, 0.38] | 0.23 | 42 | -1.10 [-1.84, -0.36] | 0.003 |
|  | No anesthesia | Zhang 2020b | - | -1.77 [-4.11, 0.57] | 0.14 | 36 | -1.07 [-1.67, -0.47] | 0.0005 |
|  |  | Zhang 2020a | - | -2.86 [-6.06, 0.33] | 0.08 | 41 | -1.09 [-1.74, -0.45] | 0.0008 |
|  | Unclear | Peruzzotti-Jametti (i) | - | -0.97 [-2.18, 0.23] | 0.11 | 18 | -1.00 [-1.53, -0.46] | 0.0002 |
|  |  | Peruzzotti-Jametti (ii) | - | -2.93 [-4.95, -0.90] | 0.005 | 43 | -1.17 [-1.88, -0.46] | 0.001 |

SMD: standardized mean difference.

**Supplement 5.** Results of leave-one-out sensitivity analysis investigating the effect of tDCS on mNSS.

|  |  |  | Subgroup analysis | | | Overall analysis | | |
| --- | --- | --- | --- | --- | --- | --- | --- | --- |
| Polarity | Subgroup | Study excluded | *I*^2^ (%) | SMD | *p* | *I*^2^ (%) | SMD | *p* |
| Anodal | - | Peruzzotti-Jametti (i) | - | 0.33 [-3.28, 3.94] | 0.86 | - | 0.33 [-3.28, 3.94] | 0.86 |
|  |  | Peruzzotti-Jametti (ii) | - | 0.87 [-1.99, 3.73] | 0.55 | - | 0.87 [-1.99, 3.73] | 0.55 |
| Cathodal | No anesthesia | - | - | - | - | - | - | - |
|  | Unclear | Peruzzotti-Jametti (i) | - | -3.53 [-7.01, -0.05] | 0.05 | 40 | -1.75 [-3.85, 0.36] | 0.10 |
|  |  | Peruzzotti-Jametti (ii) | - | -3.26 [-5.97, -0.55] | 0.02 | 51 | -1.82 [-3.82, 0.17] | 0.07 |

SMD: standardized mean difference.
